# Antioxidant Rich Potato Improves Arterial Stiffness in Healthy Adults

**DOI:** 10.1007/s11130-018-0673-2

**Published:** 2018-06-26

**Authors:** C. Tsang, N. F. Smail, S. Almoosawi, G. J. M. McDougall, E. A. S. Al-Dujaili

**Affiliations:** 1 Faculty of Health and Social Care, Edge Hill University, St Helens Road, Ormskirk, Lancashire, UK; 2 Department of Dietetics, Nutrition and Biological Sciences, Queen Margaret University, Edinburgh, UK; 3 Brain, Performance & Nutrition Research Centre, Northumbria University, Newcastle-upon-Tyne, UK; 4 Environmental and Biochemical Sciences, The James Hutton Institute, Invergowrie, Dundee, UK; 5 Centre for Cardiovascular Research, Queens Medical Research Institute, The University of Edinburgh, Edinburgh, UK

**Keywords:** Anthocyanins, Polyphenols, Cardiovascular disease, Arterial stiffness, Pulse wave velocity

## Abstract

Arterial stiffness is an emerging risk factor for cardiovascular disease and dietary anthocyanins may be important in mediating vascular tone. The present study investigated the effect of consumption of an anthocyanin-rich potato, Purple Majesty on arterial stiffness measured as pulse wave velocity in 14 healthy male and female adults. Participants consumed 200 g/day of cooked purple potato containing 288 mg anthocyanins, or a white potato containing negligible anthocyanins for 14 days, separated by a 7-day washout period. Non-invasive assessment of vascular tone by pulse wave velocity was determined in addition to systolic and diastolic blood pressure, high-density lipoproteins, low-density lipoproteins, triglycerides, glucose, insulin and C-reactive protein. Pulse wave velocity was significantly reduced (*p* = 0.001) following Purple Majesty consumption for 14-days. There were no significant changes with any other clinical parameter measured, and no changes following white potato consumption. The findings from this short-term study indicate a potential effect of Purple Majesty consumption on arterial stiffness.

**Electronic supplementary material:**

The online version of this article (10.1007/s11130-018-0673-2) contains supplementary material, which is available to authorized users.

## Introduction

Arterial stiffness (AS) is a critical precursor and strong independent risk factor for cardiovascular disease (CVD) contributing to the development of hypertension, left ventricular hypertrophy, myocardial infarction, and congestive heart failure [1, 2]. AS is a reversible process and certain dietary factors may be important in improving vascular tone [3]. Polyphenols are a diverse and heterogeneous group of secondary plant metabolites commonly classified as flavonoids, phenolic acids, stilbenes and lignans [4]. Previous studies indicate their ability to mitigate cardiovascular risk factors [5] and anthocyanins may be particularly cardio protective in this regard. As water-soluble pigments, they are responsible for the blue, red and purple colouration of many fruits and vegetables; with red wine and berries contributing a major source of anthocyanins in the UK diet [6]. Anthocyanins mediate the vascular endothelium by modulating nitric oxide bioavailability, regulating inflammatory signalling pathways, and protecting endothelial cells against oxidative stress [7–9]. Potato (*Solanum tuberosum*) is one of the most versatile and high-yielding carbohydrate-rich crops with a widespread commercial presence. They provide a valuable source of dietary antioxidants because of their high consumption rates and include ascorbic acid, carotenoids and tocopherols, with the most prevalent being phenolics; chlorogenic acid (CGA), gallic acid, protocatechuic acid and caffeic acid [10, 11]. Natural varietal cultivars include purple potatoes, which also contain anthocyanins throughout their skin, flesh and tubers; mainly as acylated glucosides of petunidin and malvidin [12]. Purple Majesty (PM), a naturally occurring variation originating from the Colorado potato-breeding program in the US, was introduced and grown in the UK for the first time. Therefore, characterisation of their phenolic components and cardio protective effects in a human study are scarce. To our knowledge, no other human study has investigated the effect of purple potatoes on pulse wave velocity (PWV). Therefore, the aim of this study was to determine the phenolic profile of PM and examine the effect on PWV in healthy male and female adults. The objective being to establish whether consumption of PM elicits a differential response compared with a white potato (WP) variety containing a similar nutrient profile albeit negligible anthocyanins.

## Materials and Methods

### Participants

Fourteen (six males and eight females) apparently healthy, non-smoking (age 20–55 years) participants were recruited through university email distribution lists. Exclusion criteria included taking medication for heart disease, hypertension, high cholesterol or diabetes, and the use of antioxidant and vitamin supplements. The study was conducted in accordance with the guidelines laid down by the Declaration of Helsinki and approved by the Research Ethics Central Committee at Queen Margaret University; Edinburgh, UK. All participants gave written consent and completed a health status questionnaire to determine their eligibility.

### Study Design

The study was a 14-day small-scale intervention of a daily intake of PM versus a WP variety. Following a 7-day run-in period, eligible participants were randomly assigned to receive a 200 g/day serving of cooked PM or WP. All potatoes were from the same batch and cooked from fresh each day (unpeeled and boiled for 15 min). Participants followed each intervention arm for 14 days, after which they were crossed-over to the next arm of the study separated by a 7-day washout period. A list of forbidden phenolic-rich foods and beverages (including green tea, black tea, coffee, red wine, dark chocolate and berries) was provided and participants were also advised to limit fruit, vegetable and potato intake over the study period. Compliance was assessed by direct observation of consumption of the meal, which was provided as a daily lunch for each participant at Queen Margaret University for the duration of the study. Food records based on a 3-day diary were used to assess compliance before, during and after each arm of the intervention.

### Purple Potato and White Potato Analysis

Potatoes from the same batch were supplied by Albert Bartlett Ltd., Airdrie, UK, and stored in the dark at 4 °C throughout the study period. The nutritional composition of the potatoes was provided by the supplier, with each 200 g serving of PM and WP containing; 154 kcal, 34.8 g carbohydrate, 4 g protein, 0.2 g fat, 2.1 g fibre and 1.6 g sugars. Potatoes were analysed prior to consumption to determine levels of total phenolics (TP), total anthocyanins (TA) and antioxidant capacity (AOX). Raw and cooked potatoes were diced and freeze-dried in vacuo overnight. Extracts were reconstituted in 50:50 methanol:water (*v/v*) and 10:90 acetone:water (*v/v*) and analysed for TP by the Folin–Ciocalteau method [13] and AOX capacity by the ferric-reducing antioxidant power assay [14], respectively. TA were determined using an adaptation of the pH shift method [15]. Osprey was selected to act as a control WP because it matched the energy and nutritional composition of PM and contained negligible anthocyanins.

### Liquid Chromatography Mass Spectrometry (LC-MS) Analysis

One hundred mg of freeze dried potato was extracted in 50% (*v/v*) aqueous acetonitrile (ACN) containing 0.1% (*v/v*) formic acid (FA) and rotated at 100 rpm for 1 h in the dark at 4 °C [16]. Samples were centrifuged (5000 *g* for 15 min at 4 °C), dried and re-suspended in 500 mL of 5% ACN/0.1% FA. A LCQ-Deca LC-MS system, comprising an autosampler, pump, photodiode array detector (PDA) and an ion-trap mass spectrometer (ThermoFinnigan, UK) was used for sample analysis. The PDA scanned discrete channels at 280, 365 and 520 nm. Samples were applied to a C18 column (Synergi Hydro C18 with polar end-capping, 2.0 × 250 mm, Phenomenex, UK) and eluted using a linear gradient of 5% ACN/0.1% FA to 40% ACN/0.1% FA over 35 min at a rate of 200 ml min^−1^. Electrospray ionization analysed the samples in positive and negative ion modes in full scan analysis followed by data-dependent MS/MS of the most intense ions using collision energies of 45%. The capillary temperature was set at 250 °C, with sheath gas at 60 psi and auxiliary gas at 15 psi. Peaks were identified by comparing their relative retention times, PDA spectra, mass to charge ratios (m/z) and MS^2^ properties with previous reports [17, 18]. Components were quantified by their UV maxima peak areas calculated using the resident software and expressed as average ± standard errors (*n* = 3). This approach is not quantitative but gives valid relative comparisons of the components between different samples or treatments.

### Measurements

Participants attended the University at the start and at the end of each treatment arm for vascular measures, anthropometric measures and blood sampling. A SECA 709 mechanical column scale was used to measure body weight to the nearest 0.1 kg and height was measured to the nearest 0.1 cm with a SECA 220 telescopic measuring rod (SECA, Birmingham, UK). Fasted blood samples were obtained by venepuncture into EDTA and lithium heparin tubes. Plasma was immediately separated by low-speed centrifugation (2500 *g* for 10 min) and stored at -80C prior to analysis. Assessment of lipid profile; high-density lipoproteins (HDL), low-density lipoproteins (LDL), and triglycerides (TAG), in addition to c-reactive protein (CRP), insulin and glucose were undertaken at the Routine Clinical Biochemistry Laboratory, Western General hospital (Edinburgh, UK), using an automated platform (Olympus, UK). Homeostasis model assessment of insulin resistance (HOMA-IR) was calculated based on published equations [19]. A validated automated A&D Medical UA-767 BP monitor (A&D medical, San Jose, CA, USA) was used to measure arterial blood pressure (BP) after a 10 min episode of horizontal resting on the subjects’ right arm using an appropriate BP cuff. Three readings were taken at 2 min intervals and mean systolic blood pressure (SBP) and diastolic blood pressure (DBP) were calculated from the second and third readings to increase the reliability of the results. Pulse wave velocity (PWV) was measured between the carotid and femoral artery (PWVcf) by means of a validated Vicorder™ device (Skidmore Medical Limited, Bristol, UK). This was performed by measuring the distance between the midpoints of two oscillometric cuffs, placed at the collar (carotid artery) and at the proximal right femur (femoral artery). A correction factor of 0.8 was used to account for the difference between the tape-measured distance between both cuffs and the reference distance, in accordance with the American Heart Association guidelines [20]. The transit time, recorded automatically, reflected the time lag between pulse wave registration at the carotid and femoral cuffs, which subsequently was divided by the distance. The mean PWVcf of three measures was recorded and the data expressed in m/s.

### Statistical Analysis

All statistical analyses were conducted using SPSS version 22.0 (SPSS Inc., Chicago, IL, USA). Baseline characteristics are presented as mean values ± standard deviations. Variables were examined for normality and skewness using Shapiro-Wilko tests, and normality was assessed with the Shapiro-Wilko test and inspection of Q-Q plots. The effect of the dietary intervention on the outcome variables was analysed using generalised linear models with Bonferroni correction after adjustment for baseline values, sex and BMI. Interaction between treatment and baseline covariates were assessed and results presented for the total group. *P*-values were two-sided, and treatment effect was considered statistically significant at *P* ≤ 0.05. Analysis of the significance of any difference observed between raw and cooked PM and WP for TP, AOX capacity and TA analysis was carried out using a paired *t*-test, and differences were considered statistically significant at *P* ≤ 0.05.

## Results and Discussion

**Fig. 1:**
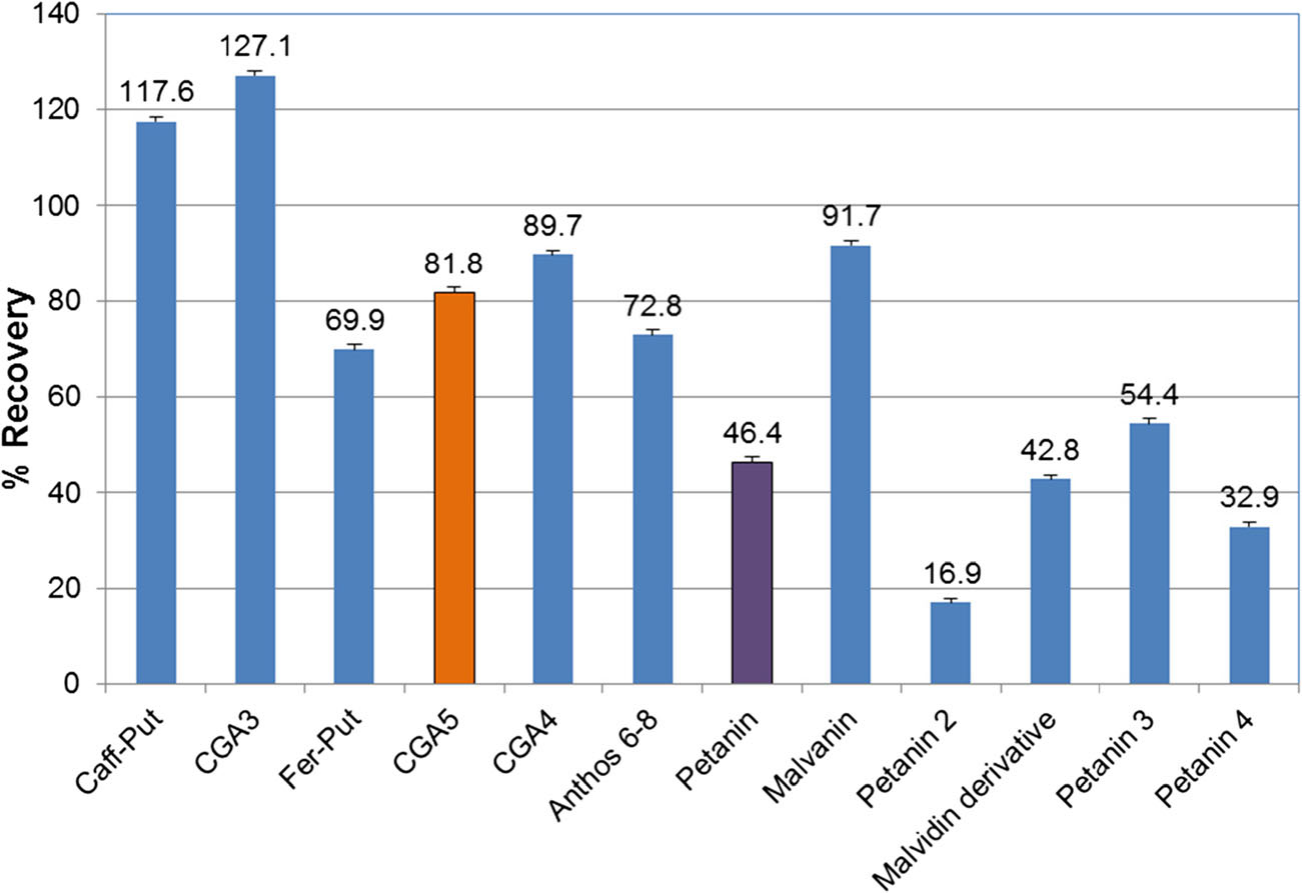
Stability of the main phenolic components in Purple Majesty after cooking. The percentage recovery of the main phenolic components was calculated as described in the material and methods. The components are fully described in Table S1. The abbreviations are as follows: Caff-Put = caffeoyl putrescine; CGA3 = chlorogenic acid isomer 3-caffeoyl quinic acid; CGA4 = 4-caffeoyl quinic acid; CGA5 = 5-caffeoyl quinic acid; Fer-Put = feruloyl putrescine; Anthos 6–8 = minor anthocyanins 6–8; Petanin 2–4 are apparent isomers of petanin; Malvinidin derivative = undefined malvidin anthocyanin. CGA5 and petanin in orange and purple respectively are the major phenolic components

Table 1 represents the levels of TP and TA, and AOX activity in raw and cooked PM and WP. TP in uncooked PM was significantly greater than WP; 1888 ± 356 and 397 ± 144 mg gallic acid equivalents (GAE) *per* kg fresh weight, respectively (*p* = 0.001). After cooking (*i.e*., boiling) TP in PM was significantly greater than WP; 1468.8 ± 361 and 228 ± 80 mg GAE per kg fresh weight, respectively (*p* = 0.001). As expected, TA was detected only in PM before and after cooking. AOX activity was significantly greater in PM before and after cooking; 11.8 ± 4.3 and 8.5 ± 3.3 mmol FeII *per* kg fresh weight, respectively compared with WP before and after cooking; 3.5 ± 2.2 and 2.8 ± 2.6 mmol FeII per kg fresh weight, respectively (*p* = 0.001). Our results were similar with those of Xu *et al.* [21] whereby domestic processing methods such as oven baking, frying, boiling and microwaving significantly reduced TP levels. In our study, potatoes were unpeeled and cooked in their skins. This may have reduced further losses as unpeeled potatoes retain greater levels of TP after boiling, baking and microwave cooking [22]. LC-MS analyses confirmed the large differences in TP and TA between PM and WP samples (see Supplementary data, Figs. S1 and S2, and Table S1) with PM containing substantial amounts of anthocyanins and WP containing no detectable anthocyanin peaks. However, PM also contained higher levels of chlorogenic acid (CGA) than WP. Although cooking PM reduced the levels of TA and TP, LC-MS analysis revealed that certain components were more affected than others (Fig. 1). The appearance of petanin (petunidin-3-rutinoside-5-glucoside acylated with *p*-coumaric acid) as the major phenolic component (and major anthocyanin) in PM agrees with previous work [23]. The presence of smaller amounts of other acylated anthocyanins was also noted previously [12]. Cooking reduced the petanin content to ~45% of the raw material. Other anthocyanins were differently affected for example; the equivalent malvidin component, called malvanin, was more stable but the apparent less abundant petanin isomers showed a range of stabilities from 15 to 54%. The CGA isomers CGA-3, 4 & 5 were the next most abundant phenolics and these were generally less affected by cooking with recoveries of 80–120%. This small overall change may reflect interchanging of forms as CGA’s undergo isomerisation reactions when heated [24]. The increase in abundance of caffeoyl putrescine may reflect increased extractability combined with relative stability.

**Table 1:** Levels of total phenolics (TP), total anthocyanins (TA) and antioxidant capacity (AOX) in raw and cooked PM and WP (mean value ± standard deviation)

Variable	PMR		PMC		WPR		WPC	
	Mean	SD	Mean	SD	Mean	SD	Mean	SD
TP ^a^	1888.6***	356.2	1468.8***	360.6	397.6	144.8	227.8	80.2
TA ^b^	1603.1	94.5	1445.1	67.3	NG	NG	NG	NG
AOX ^c^	11.7***	4.3	8.5***	3.3	3.5	2.2	2.8	2.6

NG: negligible; PMR: Purple Majesty raw; PMC: Purple Majesty cooked; WPR: white potato raw; WPC: white potato cooked. ^a^ mg gallic acid equivalent *per* kg fresh weight, ^b^ mg cyanidin-3-glucoside *per* kg fresh weight, ^c^ mmol Fe II *per* kg fresh weight cyanidin-3-glucoside equivalent *per* kg fresh weight. Statistical significance based on a paired t-test, ***p < 0.001

Tables 2 and 3 show the effect of PM and WP consumption on various risk parameters of CVD in 14 apparently healthy Caucasian non-smoking participants (six males and eight females). Participants were aged between 20 and 55 years (mean age: 33.5 ± 10.8) with a BMI between 19.4 and 31.2 kg/m^2^ (mean BMI: 22.82 ± 3.10 kg/m^2^) of which two were overweight (BMI ≥25 kg/m^2^) and one was classified as obese (BMI ≥30 kg/m^2^). No changes were observed in body weight (kg) or BMI (kg/m^2^), and there were no differences in dietary fat, carbohydrate, protein or total energy intake (data not shown) in comparison with the initial and follow-up periods. Consumption of 200 g/d of cooked PM containing 289 mg of TA for a period of 14 days improved PWV (baseline: 6.492 ± 0.5; after PM: 6.213 ± 0.5; after WP: 6.616 ± 0.6) as indicated by the statistically significant 0.3 m/s reduction in PWV (*p* = 0.001). However, unlike previous studies we did not find any changes in SBP or DBP, or any other clinical parameter associated with cardiovascular risk. One possible explanation for this could have been due to the timing of our BP measures which occurred the following morning after PM consumption. Some evidence indicates that anthocyanins transiently reduce BP returning to baseline by 4 h. Keane *et al.* [25] observed reductions in SBP over a period of 3 h post consumption of a tart cherry juice, with values returning to those similar of controls by 8 h. Therefore, any vasomodulatory effect from PM could have been missed following an overnight fast, and it is not clear at this juncture whether acute improvement in measures of vascular function persists beyond 10–12 h. Improvements in PWV without any changes in BP (or any other clinical parameter associated with cardiovascular risk) have been reported following interventions with cranberry juice and red clover isoflavones [26, 27]. Although BP is a major determinant of AS, PWV is considered an independent predictor of CVD. A recent meta-analysis including 17,635 patients from 16 studies concluded that PWV could help to identify high-risk populations, especially those not modified by hypertension, diabetes, sex or smoking status [28]. There is some evidence that CGA could also be responsible for the observed effects. Phenolic acids have demonstrated ability to modulate vascular smooth muscle cell activity *in vitro* [29] and hippuric acid and CGA have been detected in urine 6 h post-ingestion of purple potatoes [30]. Evidence of the presence of ferulic acid in serum up to 30 h post-ingestion of a radiolabelled cyanidin-3-glucoside (C3G) has also been identified [31]. However further studies are necessary to elucidate their mechanisms involved. There were several limitations to our study; we did not measure baseline levels of our parameters before the start of the second arm of the intervention period. This would have accounted for any potential changes in our baseline measurements and should be included in future studies. This was a small-scale study involving a small number of participants classified as normotensive and it is possible that any association, particularly with regard to BP lowering effect would have been considerably more pronounced in a hypertensive population [32]. The duration of the study was short (*i.e*., 14 days) and future studies should focus on longer-term studies to determine any possible changes particularly with regard to lipid parameters. Nonetheless, our small-scale study provided a detailed characterisation of the main phenolics derived from PM, introduced and grown in the UK for the first time, in comparison to a standard WP on the market. To our knowledge this is the first time a study has investigated the effect of PM on PWV in human participants.

**Table 2:** Body weight, body mass index (BMI), pulse wave velocity (PWV) and systolic (SBP) and diastolic blood pressure (DBP) measurements, at baseline and following 14 days of PM and WP consumption (mean value ± standard deviation)

Variable	Baseline		After PM		After WP	
	Mean	SD	Mean	SD	Mean	SD
Body weight (kg)	66.9	11.0	67.1	11.1	67.0	10.7
BMI (kg/m^2^)	22.7	3.1	22.8	3.1	22.8	3.1
PWV (msec)	6.5	0.5	6.2***	0.5	6.6	0.6
SBP (mmHg)	115.5	11.8	114.5	3.9	115.0	11.8
DBP (mmHg)	70.1	8.6	71.6	10.6	69.3	9.8

BMI: Body mass index; SBP: Systolic blood pressure; DBP: Diastolic blood pressure; PWV: Pulse wave velocity. *P*-values were two-sided, and treatment effect was considered statistically significant ***p < 0.001

**Table 3:** Plasma cholesterol and lipid profile, glucose, insulin, HOMA-IR and CRP measurements at baseline and following 14 days of PM and WP (mean value ± standard deviation)

Variable	Baseline		After PM		After WP	
	Mean	SD	Mean	SD	Mean	SD
TAG	0.9	0.5	0.8	0.3	0.8	0.3
TC	4.6	1.3	4.2	1.1	4.3	0.9
LDL-C	2.7	0.9	2.4	0.9	2.4	0.8
HDL-C	1.5	0.3	1.5	0.3	1.5	0.3
TC:HDL-C	3.2	0.5	2.9	0.9	2.9	0.9
CRP	5.0	0.4	5.1	0.5	5.0	0.5
Glucose	4.8	0.3	4.6	0.5	4.8	0.4
Insulin	6.2	4.1	6.4	5.5	8.4	6.4
HOMA-IR	1.3	0.9	1.3	1.1	1.8	1.5

TAG: triglycerides (mmol/L); TC: total cholesterol (mmol/L); HDL-C: HDL-cholesterol (mmol/L); LDL-C: LDL-cholesterol (mmol/L); TC:HDL-C: total cholesterol: HDL-cholesterol ratio (mmol/L); CRP: c-reactive protein; Glucose (mmol/L); Insulin (mmol/L); HOMA-IR: homeostasis model assessment of insulin resistance

## Conclusion

Consumption of PM significantly improved PWV in apparently healthy subjects without any other changes in cardiovascular markers. Future studies are warranted to validate these findings in longer-term studies, and in populations at risk of CVD to interpret their precise role as potential cardio-protective agents. PM potatoes are relatively inexpensive and commercially available in the UK and could therefore provide a valuable contribution of dietary antioxidants as part of a healthy balanced diet.

## Electronic Supplementary Material

ESM 1 (DOCX 286 kb)

ESM 2 (DOCX 273 kb)

ESM 3 (DOCX 123 kb)

## References

[1] Safar ME, London GM (2000). Therapeutic studies and arterial stiffness in hypertension: recommendations of the European Society of Hypertension. The Clinical Committee of Arterial Structure and Function. J Hypertension.

[2] Hodes RJ, Lakatta EG, McNeil CT (1995). Another modifiable risk factor for cardiovascular disease? Some evidence points to arterial stiffness. J Am Geriatr Soc.

[3] Pase MP, Grima NA, Sarris J (2011). The effects of dietary and nutrient interventions on arterial stiffness: a systematic review. Am J Clin Nutr.

[4] Manach C, Scalbert A, Morand C (2004). Polyphenols: food sources and bioavailability. Am J Clin Nutr.

[5] McCullough ML, Peterson JJ, Patel R (2012). Flavonoid intake and cardiovascular disease mortality in a prospective cohort of US adults. Am J Clin Nutr.

[6] Jennings A, Welch AA, Fairweather-Tait SJ (2012). Higher anthocyanin intake is associated with lower arterial stiffness and central blood pressure in women. Am J Clin Nutr.

[7] Wallace TC (2011). Anthocyanins in cardiovascular disease. Adv Nutr.

[8] Youdim KA, Martin A, Joseph JA (2000). Incorporation of the elderberry anthocyanins by endothelial cells increases protection against oxidative stress. Free Rad Biol Med.

[9] Serraino I, Dugo L (2003). Protective effects of cyanidin-3-o-glucoside from blackberry extract against peroxynitrite-induced endothelial dysfunction and vascular failure. Life Sci.

[10] Hejtmánková K, Pivec V, Trnková E (2009). Quality of coloured varieties of potatoes.. Czech J Food Sci.

[11] Brown CR, Culley D, Bonierbale M, Amoros W (2007) Anthocyanin, carotenoid content, and antioxidant values in native South American potato cultivars. HortScience 42:1733–1736

[12] Brown CR, Wrolstad R, Durst R et al (2003) Breeding studies in potatoes containing high concentrations of anthocyanins. Am J Pot Res 80:241–249

[13] Singleton VL, Rossi JA (1965) Colorimetry of total phenolics with phosphomolybdic-phosphotungstic acid reagents. Am J Enol Vitic 16(3):144–158

[14] Benzie IFF, Strain JJ (1996). Ferric reducing ability of plasma (FRAP) as a measure of antioxidant power: The FRAP assay. Anal Biochem.

[15] Ribereau-Gayon P, Stonestreet E (1965) Le dosage des anthocyanes dans les vines rouges. Bull Soc Chim 9:2649–2652 5848688

[16] Stushnoff C (2010). Flavonoid profiling and transcriptome analysis reveals new gene–metabolite correlations in tubers of Solanum tuberosum L. J Exp Botany.

[17] Fossen T, Andersen OM (2003) Anthocyanins from red onion, Allium cepa, with novel aglycone. Phytochemistry 62:1217–1220 10.1016/s0031-9422(02)00746-x 12648539

[18] Eichhorn S, Winterhalter P (2005) Anthocyanins from pigmented potato (*Solanum tuberosum* L.) varieties. Food Res Int 38(S8–9):943–948

[19] Matthews DR, Hosker JP, Rudenski AS (1985). Homeostasis model assessment: insulin resistance and beta-cell function from fasting plasma glucose and insulin concentrations in man. Diabetologia.

[20] Townsend RR, Wilkinson IB, Schiffrin C et al (2015) Recommendations for improving and standardizing vascular research on arterial stiffness. A scientific statement from the American Heart Association. Hypertension 66(3):698–722 10.1161/HYP.0000000000000033 PMC4587661 26160955

[21] Xu X, Li W, Lu Z (2009). Phenolic content, composition, antioxidant activity, and their changes during domestic cooking of potatoes. J Agric Food Chem.

[22] Tudela JA, Cantos E, Espín JC (2002). Induction of antioxidant flavonol biosynthesis in fresh-cut potatoes. Effect of domestic cooking. J Agric Food Chem.

[23] Stushnoff C, Holm D, Thompson MD et al (2008) Antioxidant properties of cultivars and selections from the Colorado Potato Breeding Program. Am J Pot Res 85:267–276

[24] Zanoelo EF, Beninca C (2009) Chemical kinetics of 5-O-caffeoylquinic acid in superheated steam: effect of isomerization on mate (*Ilex paraguariensis*) manufacturing. J Agric Food Chem 57:11564–11569 10.1021/jf903388a 19938862

[25] Keane KM, George TW, Constantinou CL et al (2016) Effects of Montmorency tart cherry (*Prunus cerasus* L.) consumption on vascular function in men with early hypertension. Am J Clin Nutr 103:1531–1539 10.3945/ajcn.115.123869 27146650

[26] Dohadwala MM, Holbrook M, Hamburg NM (2011). Effects of cranberry juice consumption on vascular function in patients with coronary artery disease. Am J Clin Nutr.

[27] Teede HJ, Dalais FS, Kotsopoulos D (2001). Dietary soy has both beneficial and potentially adverse cardiovascular effects: a placebo-controlled study in men and postmenopausal women. J Clin Endocrinol Metab.

[28] Vlachopoulos C, Aznaouridis K, Stefanadis C (2010). Prediction of cardiovascular events and all-cause mortality with arterial stiffness: a systematic review and meta-analysis. J Am Coll Cardiol.

[29] Keane KM, Lodge JK, Constantinou CL et al (2016) Phytochemical uptake following human consumption of tart Montmorency cherry (L. *Prunus cerasus*) and influence of phenolic acids on vascular smooth muscle cells *in vitro*. Eur J Nutr 55(4):1695–1705 10.1007/s00394-015-0988-9 26163338

[30] Tsang C, Smail NF, McDougall GJM (2015). Bioavailability and urinary excretion of phenolic derived metabolites after acute consumption of Purple Majesty potato in humans. EC Nutr.

[31] Ferrars RM, Czank C, Zhang Q (2014). The pharmacokinetics of anthocyanins and their metabolites in humans. Br J Pharmacol.

[32] Kapil V, Milsom AB, Okorie M, Maleki-Toyserkani S, Akram F, Rehman F, Arghandawi S, Pearl V, Benjamin N, Loukogeorgakis S (2010). Inorganic nitrate supplementation lowers blood pressure in humans role for nitrite-derived NO. Hypertension.

