# Supplementary material for: Antioxidant Rich Potato Improves Arterial Stiffness in Healthy Adults
Source: Plant Foods Hum Nutr. 2018 Jun 26;73(3):203–8. doi: 10.1007/s11130-018-0673-2 (PMC6096904; doi:10.1007/s11130-018-0673-2)
Supplement: Supplementary file 2 — (DOCX 273 kb) [file 11130_2018_673_MOESM2_ESM.docx]

0

5

10

15

20

25

30

Time (min)

0

5

10

15

20

25

30

35

40

45

50

55

60

65

70

75

80

85

90

95

100

Relative Absorbance

**9**

**4**

**11**

**1**

**12**

**14**

**6-8**

**3**

**5**

**2**

FSD = 2.10^e^6

**A = Purple Majesty**

**B = White potato**

**13**

**10**

Supplementary Figure S2. Comparison of Purple Majesty and white potato (Osprey). Peaks refer to Table S1.
