# Supplementary figures and images for: Antioxidant Rich Potato Improves Arterial Stiffness in Healthy Adults
Source: Plant Foods Hum Nutr. 2018 Jun 26;73(3):203–8. doi: 10.1007/s11130-018-0673-2 (PMC6096904; doi:10.1007/s11130-018-0673-2)

**Supplementary Table S1.** Components in raw and cooked Purple Majesty (see Figures S1 and S2).


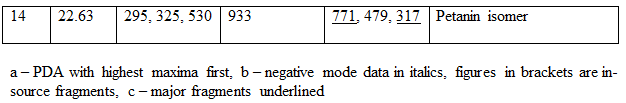
**
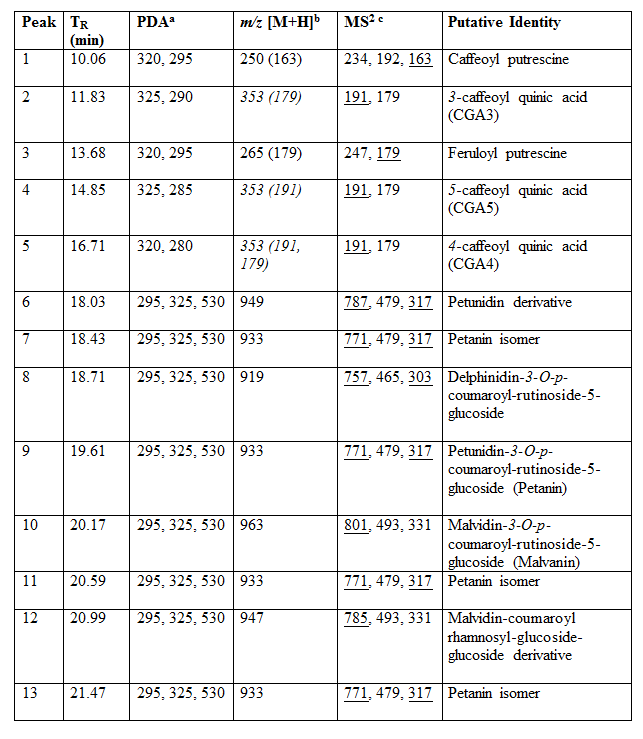
**

Supplement: Supplementary file 3 — (DOCX 123 kb) [file 11130_2018_673_MOESM3_ESM.docx]
